# Reference Intervals for Common Biochemistry and Hematology Parameters in Early Pregnancy—A Prospective Study

**DOI:** 10.3390/diagnostics15040415

**Published:** 2025-02-08

**Authors:** Vesna Šupak-Smolčić, Lucija Franin, Dragana Antončić, Sabina Matejčić, Iva Vrdoljak-Colo, Sonja Homar, Mihovil Horvat, Lidija Bilić-Zulle

**Affiliations:** 1 Clinical Department of Laboratory Diagnostics, Clinical Hospital Centre Rijeka, 51000 Rijeka, Croatia; lucija.franin@uniri.hr (L.F.); dragana.antoncic@uniri.hr (D.A.); sabina.matejcic@gmail.com (S.M.); ivacolo@gmail.com (I.V.-C.); sonja.homar@gmail.com (S.H.); mihovil.horvat@uniri.hr (M.H.); lidija.bilic.zulle@uniri.hr (L.B.-Z.); 2 Department of Clinical Laboratory Diagnostics, Faculty of Medicine, University of Rijeka, 51000 Rijeka, Croatia; 3 Department of Biomedical Informatics, Faculty of Medicine, University of Rijeka, 51000 Rijeka, Croatia

**Keywords:** clinical laboratory tests, pregnancy, reference change value, reference values

## Abstract

**Background**: The aim of this study was the determination of reference values for the common laboratory parameters in early pregnancy using a direct method and to assess their clinically significant difference, which was compared to the reference intervals for non-pregnant women with respect to the reference change value (RCV). **Methods**: This study was conducted from September 2022 to December 2023 at the Clinical Department of Laboratory Diagnostics, Clinical Hospital Centre RIJEKA, Croatia. The inclusion criteria were as follows: age ≥ 18 years, singleton pregnancy, normal ultrasound examination, and prenatal screening. The exclusion criteria were as follows: recent illness, pregnancy-related complications, medically assisted reproduction, and medication use. The reference intervals were established using the non-parametric percentile method according to the CLSI EP28-A3c recommendation. The reference values were compared to those of non-pregnant women and judged against RCV values based on biological variation. Additionally, we tested the influence of food consumption and oral supplements. **Results**: The data of 299 participants were included in the study. Laboratory tests whose changes are clinically relevant lower in early pregnancy are as follows: hemoglobin, MCV, hematocrit, MCH, urea, creatinine, albumin, alkaline phosphatase, lactate dehydrogenase, sodium, and magnesium. The clinically relevant higher values are as follows: RDW, total leukocyte count, neutrophil granulocytes, monocytes, CRP, total cholesterol, triglycerides, and amylase. UIBC has a wider reference range. The consumption of food and supplements has no clinically significant influence in relation to the RCV. **Conclusions**: Establishing reference intervals in pregnancy remains a challenge due to the metabolic changes during pregnancy, as well as its clinical significance.

## 1. Introduction

Pregnancy is a relatively short time period that is followed by intensive metabolic changes. These changes are also reflected in the metabolic products in the blood, which are crucial for the diagnosis of many pregnancy-related or non-pregnancy-related diseases. The reference values of laboratory parameters in pregnancy differ from the reference values of non-pregnant women, which has been confirmed by numerous studies [1,2,3,4]. The possibilities of implementing such reference intervals to laboratory reports of pregnant women are limited and therefore reference values for pregnancy are rarely given on laboratory reports [3]. Often, the laboratory results of pregnant women are given with reference values for non-pregnant women, which can lead to a misinterpretation of the results, additional diagnostic procedures, and unnecessary stress for the pregnant woman. Reference values for laboratory tests are established in accordance with the Clinical and Laboratory Standards Institute (CLSI) Recommendation EP28-A3c: Defining, Establishing, and Verifying reference intervals in the clinical laboratory [5]. Data can be collected prospectively (direct method) or retrospectively by analyzing data from the laboratory database (indirect method). Both methods have been used in previously published studies to determine reference values in pregnancy [1,2,3,4]. Furthermore, the CLSI recommendation points out that each laboratory should derive reference ranges specific to the population it serves or at least verify the usefulness of published reference intervals. The most common limitation in establishing reference intervals in pregnancy is the continuum of metabolic changes during pregnancy. Therefore, the division of reference intervals into pregnancy phases (usually trimesters) is very complex [3]. Furthermore, the established reference intervals should be evaluated regarding the most important metabolic changes in pregnancy and their clinical significance for the detection and differentiation of physiological and disease-related changes in pregnancy. The resulting reference values are usually compared with reference values for non-pregnant women. Groenendijk et al. have shown that most of the established reference values for pregnant women are statistically significantly different from the reference values for non-pregnant women [4]. However, a statistically significant difference is not the same as a clinically significant difference. Due to the natural fluctuations of the blood components, the changes in the measured laboratory parameters depend on the biological variation of the respective parameters and on the variation of the analytical method. These two variables are combined in the calculation of a clinically significant change (the reference change value, RCV), which represents the maximum percentage difference between two measurements as a result of normal metabolism [6]. A difference that exceeds the predicted RCV represents a clinically significant change and indicates an event that should be considered diagnostically and treated. The aim of this work is to determine reference values for the most common laboratory parameters in early pregnancy using a direct method and to determine their clinically significant difference compared to the reference intervals for non-pregnant women.

## 2. Materials and Methods

This prospective study was conducted in the Clinical Department of Laboratory Diagnostics of the Hospital Centre Rijeka, Croatia, from September 2022 to December 2023. Pregnant women were recruited during their laboratory visit for non-invasive prenatal screening between the 11th and 14th week of gestation. The inclusion criteria were as follows: age ≥ 18 years, singleton pregnancy, and normal last ultrasound findings and prenatal screening results. The exclusion criteria were as follows: recent illness, pregnancy-related complications, medically assisted reproduction, and chronic medication use. After the informed consent interview and confirmation that they were eligible for the study, all participants completed a questionnaire about their habits and current health status and signed the informed consent form. The questionnaire contained the following questions: demographic data, height and weight at the last examination, fasting status (fasting is not mandatory), date of last menstrual period according to which the week of pregnancy is calculated (unless otherwise indicated by the participant’s gynecologist), alcohol and tobacco consumption during pregnancy, intake of dietary supplements during pregnancy, special dietary habits, acute illnesses in the last month, regular physical activity and sports, last blood pressure measurement, and number of previous pregnancies and births.

Two tubes of blood were drawn from each participant according to the current phlebotomy recommendations [7], one purple K2EDTA tube (Vacuette, Greiner bio-one, REF 454246) for the complete blood count and erythrocyte sedimentation rate and one red tube with a clot activator and gel barrier (Vacuette, Greiner bio-one, REF 455071) for biochemistry analyses. All tubes were processed according to the manufacturer’s recommendations and the laboratory’s standard procedures. The samples were analyzed on the day of collection.

The complete blood count was determined using the Beckman Coulter DxH800 (Beckman Coulter, Brea, CA, USA), and the erythrocyte sedimentation rate was determined using the iSED (Alcor Scientific, Smithfield, RI, USA). The serum sample was used for the following analyses: urea, creatinine, uric acid, albumin, total protein, C-reactive protein (CRP), total cholesterol, triglycerides, lactate dehydrogenase (LD), creatine kinase (CK), aspartate aminotransferase (AST), alanine aminotransferase (ALT), alkaline phosphatase (AP), gamma-glutamyl transferase (GGT), alpha-amylase (AMY), total and conjugated bilirubin, iron, unsaturated iron binding capacity (UIBC), sodium, potassium, chloride, calcium, magnesium, and inorganic phosphate. These were analyzed using the Roche Cobas 6000 c501 (Roche Diagnostics, Mannheim, Germany). Ferritin and HDL cholesterol were analyzed using the Beckman Coulter AU480 (Beckman Coulter, Brea, CA, USA). LDL cholesterol was calculated according to the Sampson equation [8]. All analytical methods and their performance data with the analytical quality specification according to the European Federation for Clinical Chemistry and Laboratory Medicine (EFLM) Biological Variation Database, except for the erythrocyte sedimentation rate (ESR), which was assessed according to Penev M et al., are listed in Table 1 [9,10].

### Statistical Analysis

The reference values were determined using the non-parametric percentile method according to the CLSI C28-A3 recommendation [5]. This method is used when the data are not normally distributed. When low and high values of a particular analyte are clinically relevant, the reference range is expressed as the central 95% of the measured values of the specific population, bounded by the lower and upper reference limits which are presented as the 2.5th and 97.5th percentiles of the data and their respective 90% confidence intervals. If only one value is of clinical relevance, e.g., a high value, then the reference limit is the 97.5th percentile of a given set of values. Before calculating the reference intervals, all data were visually inspected and tested for outliers according to Reed et al. [11].

The determined reference values were compared with the reference values recommended by the Croatian Chamber of Medical Biochemists for non-pregnant women, which are harmonized for all laboratories in Croatia [12]. For each reference value, the clinically significant change was calculated as the percentage difference between the reference value for pregnant and non-pregnant women. This percentage was then compared with the reference change value (RCV) calculated according to the following equation [6]:

RCV = 2^1/2^ × Z × (CV_A_^2^ + CV_I_^2^)^1/2^, (1)

where Z is 1.96 for the analytes whose increase and decrease (change) are considered equally (two-sided), and Z is 1.65 for the analytes whose one-sided change is considered, regardless of whether it is an increase or decrease. The chosen Z value represents the standard deviation for the desired probability of *p* < 0.05. The CV_A_ is the analytical imprecision calculated from the daily internal quality control results of our laboratory (see Table 1), and CV_I_ is the within-subject biological variation reported in the EFLM biological variation database [9]. The CV_A_ was assessed using the quality specifications for desirable imprecision given in the same EFLM database.

To exclude the influence of supplement and food consumption, we compared all relevant laboratory tests between the women who were taking supplements and/or not fasting and those who were not taking supplements or were fasting. The distribution of the data was tested for normality using the Kolmogorov–Smirnov test. The normally distributed data were compared using the *t*-test, and the non-normally distributed data were tested using the Mann–Whitney test. The statistical significance was set at *p* < 0.05.

All statistical computations were performed using statistical software MedCalc version 23.0.2 (MedCalc Software, Ostend, Belgium, license of the University of Rijeka, Faculty of Medicine).

Each participant signed an informed consent form after the initial interview. This study was approved by the Ethics Committee of the Clinical Hospital Centre Rijeka (ethical approval number: Klasa: 003-05/22-1/82; Ur.br: 2170-29-02/1-22-2; date: 19 August 2022).

## 3. Results

A total of 312 pregnant women from the 11th to 14th week of pregnancy were initially recruited for the study, of whom 13 were excluded subsequently. Two of them were later found to be pregnant due to a medically assisted reproduction procedure, two had a fetus with trisomy 21, three lost their pregnancy shortly after recruitment, one was on chronic thyroxine therapy although she had not declared this at recruitment, three had complications in later pregnancy or at delivery, and two were excluded due to extreme BMI (>40 kg/m^2^). Finally, 299 pregnant women were included in the study.

The median (minimum–maximum) age of study participants was 30 (18–41) years. The median (interquartile range) body mass index was 23.5 (21.1–26.8) kg/m^2^, the median systolic blood pressure was 110 (105–120) mmHg, and the diastolic blood pressure median was 65 (60–70) mmHg.

Out of 299 women, 198 (66%) were taking oral supplements (folic acid, other vitamins, magnesium, and iron). As fasting was not required for participation in the study, 87 (29%) blood samples were collected in a fasting state. The results of the influence of dietary supplements and food on the laboratory results are shown in Table 2. Out of 299 women, 202 (68%) gave birth vaginally, 66 (22%) gave birth by cesarean section, and we have no data for 31 (10%) participants as they gave birth in another facility. All women with available delivery data gave birth to a healthy child.

The calculated reference intervals are shown in Table 3. The Reed test for outliers showed only two outliers in our data set. Both were excluded from the calculation. The outliers were one CK and one ALT measurement found by chance, and pregnancy-related pathology was later ruled out by medical examination.

Laboratory tests whose change is clinically relevant in the first trimester of pregnancy compared to non-pregnant women are the hemoglobin concentration and the MCV value, which are lower in pregnancy. Consequently, all calculated parameters derived from these two are also clinically significantly changed in the first trimester of pregnancy, including hematocrit and MCH. The total leukocyte count, the number of neutrophil granulocytes, and the RDW are clinically significantly higher than in non-pregnant women. The lower reference limit for monocytes is also higher in the first trimester. Of all biochemistry tests, both reference limits are clinically relevantly lower for creatinine and AP, and the upper reference limit is lower for urea, albumin, LD, sodium, and magnesium. CRP, total cholesterol, and triglycerides are clinically relevantly elevated in the first trimester of pregnancy. The enzymes AST, ALT, GGT, and AMY have a decreased lower reference limit, but this has no clinical significance. The UIBC value has a wider reference range in the first trimester of pregnancy than in non-pregnant women.

According to our results, the intake of food supplements has an influence on the total leukocyte count, as pregnant women who take food supplements have a lower leukocyte count in comparison with pregnant women who do not take food supplements (8.58 ± 1.98 × 10^9^/L vs. 9.07 ± 2.16 × 10^9^/L, *p* = 0.049) (shown in Table 2). The consumption of food before phlebotomy affects the measurement of the ESR (19 (12–26) vs. 16 (11–23) *p* = 0.033), LDL cholesterol (2.4 (1.9–2.8) mmol/L vs. 2.1 (1.8–2.5) mmol/L *p* = 0.027), total bilirubin (6 (4–8) µmol/L vs. 5 (3–7) µmol/L *p* = 0.013), and conjugated bilirubin (2 (2–3) µmol/L vs. 2 (1–2) µmol/L *p* = 0.024), which are higher in fasting individuals. Triglycerides are slightly elevated in non-fasting subjects (1.0 (0.8–1.5) vs. 1.2 (0.9–1.5) *p* = 0.017) (shown in Table 2). However, none of these statistically significant differences are clinically significant in relation to the reference change value (RCV). The calculated percentage differences of those parameters when compared to the RCV revealed the following results: leukocyte 5.40% vs. 31.01%, ESR 18.75% vs. 95.49%, triglycerides 16.67% vs. 46.34%, LDL cholesterol 14.29% vs. 19.28%, total bilirubin 20.00% vs. 56.45%, and conjugated bilirubin 0% vs. 33.32%.

## 4. Discussion

Changes in laboratory parameters are already visible in the first trimester of pregnancy and are the result of numerous adaptive changes in maternal metabolism. In our study, the reference values of numerous biochemical and hematological parameters in the first trimester of pregnancy that apply to our population were successfully determined. Of the laboratory parameters studied, clinically significant changes were found in hemoglobin, MCV, and RDW in pregnant women compared to non-pregnant women, and consequently also in hematocrit and MCH, which are calculated from hemoglobin and MCV. The changes during pregnancy, such as an increase in plasma volume by 25–80%, are mainly reflected in lower levels of hemoglobin, hematocrit, and red blood cell count [1,4,13,14]. Our results are consistent with previously published research, but the change in erythrocyte reference values in early pregnancy is not clinically significant. It is possible that in early pregnancy the increased erythropoietin synthesis and its effect on red cell production still compensates for the significant decrease in the total red cell count due to the increase in plasma volume. However, some studies show that the effect of erythropoietin on red cell mass is reduced in the first trimester of pregnancy [15]. Our study has shown that the RDW is higher in pregnancy and could therefore be a sign of the increased mobilization of erythrocytes from the bone marrow. An increased need for erythrocyte synthesis also requires a higher iron influx. Our results show that there is no difference in blood iron or ferritin concentrations in the first trimester compared to non-pregnant women, and we demonstrated no influence of supplement and food intake on blood iron and ferritin concentrations. Our results show that pregnant women have a higher unsaturated iron binding capacity in the blood already in the first trimester compared to non-pregnant women, which can also be explained by an adaptation mechanism to the increased iron consumption in the early phase of pregnancy, as confirmed by Yang et al. [16]. Other studies have measured transferrin directly and have not confirmed a significantly higher transferrin concentration in the first trimester of pregnancy, but have not investigated the unsaturated iron binding capacity [1,4]. The reference values for red blood cell parameters in pregnancy are of great importance for public health, as the World Health Organization defines anemia in pregnancy as a hemoglobin level below 110 g/L, and in terms of early detection of pregnant women for iron supplementation [17]. Our study found that the lower limit of the reference interval for hemoglobin in the first trimester is 109 g/L, which is consistent with other studies [3,14]. Recommendations for the diagnosis and treatment of iron deficiency anemia in pregnancy are inconsistent, and blood cell parameter results have been shown to vary by population. Therefore, a larger number of studies on laboratory parameters in pregnancy will certainly provide more data for better-quality meta-analyses stratified by ethnicity [18,19].

Other blood parameters that changed clinically significantly according to our study are the total leukocyte count, neutrophil granulocytes, and the lower limit of the reference interval for monocytes. These results are consistent with previously published results and confirm the increase in total leukocyte count at the expense of neutrophils in the first trimester of pregnancy. The reference intervals determined in this study confirm the previously published results of Barakauskas et al. who covered the entire gestational period and concluded that the increase in leukocytes at the expense of neutrophils is most pronounced in the first trimester of pregnancy and remains elevated until the end of pregnancy [3]. Dokree et al. have shown that the elevated leukocyte levels do not change significantly from the 8th week of gestation until delivery and that the reference values for leukocytes do not need to be stratified by trimester [20]. Other leukocyte subpopulations are not clinically significantly altered in the first trimester, which is consistent with other studies [3,20]. However, in our study, we identified a slightly higher lower limit of the reference interval for monocytes. Research shows that the first trimester of pregnancy is a pro-inflammatory phase of pregnancy that allows for the implantation and maintenance of the blastocyst and placenta. Briefly and simply stated, the cells that enable maternal immunologic tolerance to fetal growth are recruited from peripheral blood leukocyte populations, primarily monocytes, neutrophils, NK cells, and T lymphocytes [21]. It is possible that the number of monocytes in the first few weeks is even higher than in non-pregnant women, but the differences in circulating cells are probably not of clinical significance according to the conclusion of Dockree et al. [20].

In relation to the inflammatory markers, the C-reactive protein is already significantly elevated in the early phase of pregnancy, which was confirmed by our results. The cut-off value for CRP determined in our study is 3.5 times higher than the value determined for the rest of the population. The literature indicates that CRP concentrations are elevated throughout pregnancy, although various studies show different dynamics of changes in CRP concentrations up to delivery, suggesting that CRP concentrations are highest in the early stages of pregnancy [1,22,23]. Such contradictory data limit the use of CRP as a marker for inflammatory conditions in pregnancy, as it is difficult to define cut-off values for clinical decision-making. Dockree et al. established reference values for CRP that apply to the entire period of pregnancy and found that concentrations do not differ significantly by the week of gestation [24]. The upper limit of the reference interval determined by Dockree et al. is 19 mg/L compared to the value of 17.2 mg/L determined in our study. In their study, they demonstrated that the diagnostic accuracy of CRP in the detection of chorioamnionitis is significantly improved when pregnancy-appropriate reference intervals are used compared to the current guidelines’ cut-off values [24]. This example illustrates once again how important it is to use appropriate reference values that are characteristic of pregnancy for laboratory findings to have diagnostic value.

The biochemistry parameters most affected by the physiological changes in early pregnancy such as a 50% increase in the glomerular filtration rate and an up to 80% increase in renal plasma flow, are urea, creatinine, and sodium, which we also confirmed in our study [25]. Although these changes are already known, they should be kept in mind when interpreting the findings when reference values for non-pregnant women are given in laboratory reports of pregnant women, in whom slightly elevated creatinine concentrations may go unnoticed as an indicator of altered renal function. The literature usually mentions 20–25% lower values for urea, creatinine, and uric acid [26]. Our study confirmed a change in urea and creatinine of more than 25% and in uric acid of about 20%, but this change in uric acid concentration in the first trimester of pregnancy did not prove to be clinically significant. The electrolyte changes in our study are clinically significant for sodium and magnesium, which are lower in the first trimester of pregnancy. The literature data suggest lower levels of all electrolytes during pregnancy, mainly due to the increased plasma volume and glomerular filtration rate. In addition, indirect electrolyte measurements in a diluted sample are sensitive to changes in serum proteins that occur during pregnancy, such as lower albumin concentrations. In our study, no clinically significant change in potassium or chloride concentrations determined by indirect potentiometry could be detected, only sodium. Furthermore, calcium is also an electrolyte that is sensitive to albumin concentration. Considering that the calcium level was not clinically significantly altered in our study, we can conclude that the influence of an altered albumin concentration in the early phase of pregnancy is not reflected in the measurement of electrolyte concentration. In addition, hyponatremia also occurs as a result of altered sensitivity to the effect of the antidiuretic hormone. Therefore, the correct interpretation of sodium findings in pregnancy is crucial, as hyponatremia is often a consequence of pre-eclampsia, which is crucial to recognize at the earliest stage [26].

According to our results, albumin is also clinically significantly lower in the first trimester of pregnancy, which is consistent with previously published studies [1,4]. The concentration of albumin in pregnancy depends on the balance between its increased synthesis in the liver and consumption as a source of amino acids for fetal growth and development, with the inevitable influence of dilution due to increased plasma volume [1]. The change in albumin concentration later in pregnancy is more pronounced, and other blood components bound to albumin also change, as already discussed in relation to electrolytes.

A clinically significant change was found for the lower limit of the reference range for AST, ALT, and GGT, which are lower in pregnancy compared to non-pregnant women. Elevated concentrations of liver enzymes have an important diagnostic value in the early detection and differential diagnosis of liver diseases in pregnancy such as intrahepatic cholestasis, HELLP syndrome, or acute fatty liver of pregnancy. However, the lower limits of the reference interval have no clinical significance, so we can conclude that there is no significant clinical difference in liver enzyme values in pregnancy compared to non-pregnant women, which was confirmed by almost identical results in the study by Groenendijk et al. [4]. The same study found very similar reference intervals for amylase, which is slightly higher in pregnant women compared to non-pregnant women. Through our study, we obtained clinically significantly lower values for alkaline phosphatase compared to non-pregnant women. Since alkaline phosphatase levels increase significantly during pregnancy at the expense of placental isoenzyme and later bone isoenzyme, alkaline phosphatase has less diagnostic significance for the diagnosis of intrahepatic cholestasis, the most common liver disease in pregnancy [27]. LDH levels are clinically significantly lower in early pregnancy compared to non-pregnant women, which is consistent with published data from the study by Groenendijk et al. and Friis Petersen et al. [4,23]. LDH is not only a marker for tumor growth but is also important for the diagnosis of preeclampsia and HELLP syndrome, and the results should be interpreted in the context of reference values adjusted for pregnant women [26,28].

Our study confirmed clinically significantly elevated levels of total cholesterol and triglycerides in early pregnancy, which is consistent with numerous published studies [1,3,4,23]. However, the reference values in our study are slightly lower than those published by Friis Petersen et al., who published a reference interval for total cholesterol of 3.0 to 8.0 mmol/L for >12 weeks of gestation [23]. Our results agree with those of Groenendijk et al. and Klajnbard et al. [1,4]. Lipids in the blood increase to very high concentrations during pregnancy to meet the energy needs of the mother and the growing fetus and serve as structural molecules and precursors of numerous bioactive molecules and hormones. High lipid concentrations are a consequence of increased insulin resistance and lipogenesis, as well as the effect of estrogen. The role of lipids as an energy source and building blocks for mother and child is undeniable, but there are also numerous complications and adverse outcomes associated with hyperlipidemia in pregnancy, such as preeclampsia, gestational diabetes mellitus, preterm delivery, intrahepatic cholestasis, and fetal growth restriction [29]. In the general population, serum lipid levels are estimated regarding the limits of clinical judgment in assessing the risk of adverse cardiovascular events. Reference values for the lipid profile are not known for the population in Croatia, as all laboratories report recommended values according to the harmonization of reference intervals [12]. Taking these values into account, we found a clinically significant change in reference values in early pregnancy. While no clinically significant change was found for LDL and HDL cholesterol, total cholesterol, and triglycerides differed significantly compared to non-pregnant women, regardless of previous food intake. Despite numerous studies and investigations on lipid changes in pregnancy, there are no defined clinical decision limits that would facilitate the interpretation of lipid profile findings and enable the prediction of adverse pregnancy outcomes [29,30,31].

A limitation of this study is the unknown outcome of pregnancy in 31 of a total of 299 pregnant women included in the calculation of the reference intervals. However, as this study only included women in early pregnancy, we assumed that the health status during this period was not influenced by possible complications during delivery. Furthermore, the results of these participants did not differ from the other results and did not represent an outlier.

In summary, of the 44 analytes tested in our study, 20 analytes showed clinically relevant changes in reference values in the first trimester of pregnancy compared to the reference intervals for non-pregnant women. In pregnancy, hemoglobin, hematocrit, MCV, MCH, urea, creatinine, albumin, alkaline phosphatase, LDH, sodium, and magnesium are lower and RDW, white blood cell count, neutrophil count, monocytes, CRP, total cholesterol, triglycerides, and amylase are higher, while UIBC has a wider reference range.

The intake of food supplements has an influence on the total leukocyte count and consumption of food affects the measurement of erythrocyte sedimentation rate, LDL cholesterol, total and conjugated bilirubin, and triglycerides. However, none of these differences are clinically significant in relation to the RCV.

Establishing reference intervals in pregnancy remains a challenge due to the constant metabolic changes during pregnancy, as well as assessing the clinical significance of individual laboratory tests for diagnoses that are unique to pregnancy. Studies showing conflicting results highlight the need for each laboratory to review the usefulness of existing reference intervals or develop their own, which is challenging in the case of pregnancy. In addition, a larger number of studies with similar results will strengthen the established reference values for a given population and allow their more reliable application, depending of course on ethnicity, climate, and lifestyle. The implementation of reference intervals in the laboratory information system so that they are available for the correct interpretation of laboratory analysis results remains a challenge.

## Figures and Tables

**Table 1:** The analytical methods used in the study and their analytical performance assessed according to the specifications of the Biological variation database [9], with the exception of the erythrocyte sedimentation rate (ESR), which was assessed according to Penev M et al. [10]. A complete blood count was performed using the Beckman Coulter DxH800 (Beckman Coulter, Brea, CA, USA), the erythrocyte sedimentation rate was assessed using the iSED (Alcor Scientific, Smithfield, RI, USA), and all biochemistry tests were determined on the Roche Cobas 6000 c501 (Roche diagnostics, Mannheim, Germany), except for ferritin and HDL cholesterol which were analyzed using the Beckman Coulter AU480 (Beckman Coulter, Brea, CA, USA).

Analyte (Unit)	Analytical Method	Laboratory’s CV_A_	Analytical Quality Specifications *		
			CVmin	CVdes	CVopt
Red blood cell count (RBC) (×10^12^/L)	Impedance (Coulter principle)	1.1	2.1	1.4	0.7
Hemoglobin (Hb) (g/L)	Photometric measurement	0.7	2.0	1.4	0.7
Hematocrit (Hct) (L/L)	Calculated (Hct = RBC × MCV)	1.4	2.1	1.4	0.7
Mean erythrocyte corpuscular volume (MCV) (fL)	Impedance (derived from RBC histogram)	1.3	0.6	0.4	0.2
Mean corpuscular hemoglobin (MCH) (pg)	Calculated (Hb/RBC)	0.9	0.5	0.3	0.2
Mean corpuscular hemoglobin concentration (MCHC) (g/L)	Calculated (Hb/Hct)	1.3	0.8	0.5	0.3
Red cell distribution width (RDW) (%)	Derived from the RBC histogram	0.9	1.3	0.8	0.4
Mean platelet corpuscular volume (MPV) (fL)	Derived from the platelet histogram	0.8	1.7	1.1	0.6
Platelet count (PLT) (×10^9^/L)	Impedance (Coulter principle)	2.3	5.5	3.6	1.8
Leukocyte count (WBC) (×10^9^/L)	Impedance (Coulter principle)	1.4	8.3	5.5	2.8
Neutrophile granulocytes (×10^9^/L)	COULTER^®^ VCSn technology	1.6	10.6	7.0	3.5
Lymphocytes (×10^9^/L)	COULTER^®^ VCSn technology	3.2	8.1	5.4	2.7
Monocytes (×10^9^/L)	COULTER^®^ VCSn technology	5.8	10.0	6.7	3.3
Eosinophile granulocytes (×10^9^/L)	COULTER^®^ VCSn technology	8.4	11.3	7.5	3.8
Basophile granulocytes (×10^9^/L)	COULTER^®^ VCSn technology	30.7	9.3	6.2	3.1
Erythrocyte sedimentation rate (ESR)	Capillary photometry	7.6	25.2	16.8	8.4
Urea (mmol/L)	Urease-GLDH (traceable to ID/MS)	1.9	10.0	6.7	3.3
Creatinine (µmol/L)	Compensated Kinetic Jaffé (traceable to ID/MS)	2.8	3.3	2.2	1.1
Uric acid (µmol/L)	Uricase-PAP (traceable to ID/MS)	1.9	6.1	4.0	2.0
Albumin (g/L)	Colorimetric with BCG (traceable to BCR470/CRM470)	1.9	1.9	1.3	0.6
Total protein (g/L)	Biuret (traceable to SRM 927)	1.5	2.0	1.3	0.7
C-reactive protein (CRP) (mg/L)	Immunoturbidimetric assay (traceable to CRM 470)	2.6	25.3	16.9	8.4
Ferritin (µg/L)	Immunoturbidimetric assay (traceable to NIBSC 94/572)	4.3	9.7	6.5	3.2
Total cholesterol (mmol/L)	CHOD-PAP (traceable to ID/MS)	2.4	3.9	2.6	1.3
Triglycerides (mmol/L)	GPO-PAP with 4-aminophenazone (traceable to ID/MS)	2.5	14.8	9.8	4.9
HDL cholesterol (mmol/L)	Homogeneous CHOD-PAP assay with anti-beta-lipoprotein-antibody (traceable to American CDC reference method)	4.1	4.3	2.9	1.4
LDL cholesterol (mmol/L)	Calculated according to Sampson	/	^†^	NA ^†^	NA ^†^
Lactate dehydrogenase (LD) (U/L)	IFCC (optical enzymatic test)	1.5	3.3	2.2	1.1
Creatin kinase (CK) (U/L)	IFCC (optical enzymatic test)	1.5	10.6	7.0	3.5
Aspartate aminotransferase (AST) (U/L)	IFCC without pyridoxal phosphate	1.7	6.4	4.3	2.1
Alanine aminotransferase (ALT) (U/L)	IFCC without pyridoxal phosphate	2.2	8.6	5.7	2.9
Alkaline phosphatase (AP) (U/L)	IFCC (enzymatic with p-nitrophenyl phosphate)	2.5	4.5	3.0	1.5
Gamma-glutamyl transferase (GGT) (U/L)	IFCC (enzymatic with L-γ-glutamyl-3-carboxy-4-nitroanilide)	1.6	6.2	4.2	2.1
Alpha amylase (AMY) (U/L)	IFCC (enzymatic with E-G_7_PNP)	1.7	4.9	3.3	1.6
Total bilirubin (µmol/L)	Diazo method (traceable to Doumas method)	2.6	15.1	10.1	5.0
Conjugated bilirubin (µmol/L)	Diazo method (traceable to Doumas method)	2.8	10.5	7.0	3.5
Iron (µmol/L)	Colorimetric assay with ferrozine (traceable to SRM 937)	2.3	20.7	13.8	6.9
Unsaturated iron binding capacity (UIBC) (µmol/L)	Colorimetric assay with ferrozine (standardized through iron assay)	3.2	NA	NA	NA
Sodium (Na) (mmol/L)	Indirect potentiometry	1.3	0.4	0.3	0.1
Potassium (K) (mmol/L)	Indirect potentiometry	1.5	2.9	1.9	1.0
Chloride (Cl) (mmol/L)	Indirect potentiometry	1.4	0.8	0.5	0.3
Calcium (Ca) (mmol/L)	Colorimetric method with 5-nitro-5′-methyl-BAPTA (traceable to SRM 956)	1.4	1.4	0.9	0.5
Magnesium (Mg) (mmol/L)	Colorimetric method with xylidyl blue (traceable to SRM 956)	1.8	2.0	1.3	0.7
Inorganic phosphate (P) (mmol/L)	Molybdate UV method (traceable to NIST)	2.2	5.8	3.9	1.9

* CVmin—minimal criteria for coefficient of variation; CVdes—desirable criteria for coefficient of variation; CVopt—optimal criteria for coefficient of variation; CV_A_—analytical coefficient of variation. ^†^ NA—Not applicable—LDL cholesterol is calculated using Sampson equation and therefore analytical quality specification for direct analytical method cannot be applied.

**Table 2:** The influence of oral nutritional supplements and fasting status on laboratory test results. The difference was tested with the *t*-test and the results are presented as the arithmetic mean and standard deviation for normally distributed data. The Mann–Whitney test was used for non-normally distributed data, which are presented as the median and interquartile range. *p* < 0.05 is considered statistically significant and statistically significant differences are shown as bold values.

Analyte (Unit) *	Non-Supplements (N = 101)	Supplements (N = 198)	*p*-Value	Non-Fasting (N = 212)	Fasting (N = 87)	*p*-Value
RBC (×10^12^/L)	4.26 ± 0.30	4.31 ± 0.28	0.176	4.31 (4.06–4.47)	4.30 (4.14–4.44)	0.484
Hemoglobin (Hb) (g/L)	124 (119–130)	126 (121–131)	0.336	125 ± 8	126 ± 8	0.176
Hematocrit (Hct) (L/L)	0.379 ± 0.022	0.383 ± 0.023	0.210	0.381 ± 0.023	0.384 ± 0.024	0.279
MCV (fL)	89.6 (87.1–91.7)	89.5 (86.4–91.7)	0.430	89.6 (86.6–91.8)	89.0 (86.7–91.4)	0.701
MCH (pg)	29.5 (28.4–30.5	29.4 (28.3–30.1)	0.222	29.4 (28.3–30.2)	29.4 (28.3–30.2)	0.905
MCHC (g/L)	328 (324–333)	328 (324–332)	0.726	328 ± 7	328 ± 6	0.343
RDW (%)	13.5 (13.1–14.0)	13.4 (13.0–13.9)	0.466	13.5 (13.1–13.9)	13.4 (13.0–13.9)	0.489
MPV (fL)	8.6 (7.9–9.3)	8.4 (7.8–9.2)	0.504	8.5 (7.9–9.2)	8.5 (7.7–9.3)	0.658
PLT (×10^9^/L)	240 (211–272)	228 (205–263)	0.074	231 (207–263)	233 (204–272)	0.925
WBC (×10^9^/L)	9.07 ± 2.16	8.58 ± 1.98	**0.049**	8.83 ± 1.98	8.52 ± 2.20	0.232
Neutrophile granulocytes (×10^9^/L)	6.61 ± 1.86	6.23 ± 1.70	0.076	6.32 (5.15–7.47)	5.95 (4.65–7.29)	0.095
Lymphocytes (×10^9^/L)	1.71 (1.44–1.95)	1.65 (1.38–1.92)	0.187	1.69 ± 0.44	1.68 ± 0.41	0.846
Monocytes (×10^9^/L)	0.55 (0.45–0.67)	0.52 (0.43–0.61)	0.082	0.52 (0.44–0.65)	0.53 (0.44–0.61)	0.919
Eosinophile granulocytes (×10^9^/L)	0.09 (0.06–0.14)	0.08 (0.05–0.14)	0.427	0.09 (0.05–0.13)	0.09 (0.06–0.16)	0.079
Basophile granulocytes (×10^9^/L)	0.03 (0.02–0.05)	0.03 (0.02–0.04)	0.214	0.03 (0.02–0.04)	0.03 (0.02–0.04)	0.975
ESR	18 (11–25)	16 (11–24)	0.268	16 (11–23)	19 (12–26)	**0.033**
Urea (mmol/L)	2.9 (2.5–3.4)	3.0 (2.6–3.5)	0.117	3.0 (2.6–3.6)	2.8 (2.5–3.4)	0.099
Creatinine (µmol/L)	49 (43–53)	49 (44–53)	0.597	49 (44–53)	49 (44–54)	0.815
Uric acid (µmol/L)	175 (155–213)	174 (148–197)	0.231	175 ± 38	185 ± 46	0.064
Albumin (g/L)	42.0 ± 1.9	42.5 ± 2.1	0.053	42.2 ± 2.1	42.4 ± 1.8	0.422
Total protein (g/L)	68 (66–70)	68 (66–70)	0.321	68 (66–70)	68 (67–70)	0.185
CRP (mg/L)	3.2 (1.6–6.9)	2.8 (1.4–5.0)	0.056	2.9 (1.5–5.5)	2.9 (1.3–6.0)	0.967
Ferritin (µg/L)	33 (21–57)	39 (23–60)	0.456	36 (22–56)	43 (22–66)	0.255
Total cholesterol (mmol/L)	4.5 (4.1–5.1)	4.5 (4.1–5.1)	0.557	4.6 (4.1–5.0)	4.5 (4.2–5.2)	0.399
Triglycerides (mmol/L)	1.2 (0.9–1.5)	1.1 (0.9–1.5)	0.351	1.2 (0.9–1.5)	1.0 (0.8–1.5)	**0.017**
HDL cholesterol (mmol/L)	1.8 (1.6–2.0)	1.9 (1.6–2.1)	0.119	1.8 (1.6–2.0)	1.8 (1.6–2.1)	0.571
LDL cholesterol (mmol/L)	2.3 (1.8–2.6)	2.1 (1.9–2.6)	0.988	2.1 (1.8–2.5)	2.4 (1.9–2.8)	**0.027**
LD (U/L)	140 (126–153)	137 (129–150)	0.701	137 (129–150)	138 (127–152)	0.917
CK (U/L)	52 (41–65)	52 (41–64)	0.975	52 (42–66)	51 (40–64)	0.333
AST (U/L)	14 (12–16)	14 (13–16)	0.834	14 (13–16)	14 (12–16)	0.862
ALT (U/L)	11 (9–15)	11 (9–15)	0.427	11 (9–15)	11 (9–14)	0.605
AP (U/L)	51 (43–61)	48 (42–56)	0.051	48 (41–58)	50 (43–59)	0.150
GGT (U/L)	11 (8–14)	10 (8–12)	0.430	10 (8–12)	10 (9–14)	0.501
AMY (U/L)	55 (46–70)	60 (49–74)	0.072	58 (48–72)	61 (47–77)	0.607
Total bilirubin (µmol/L)	5 (3–6)	5 (4–7)	0.356	5 (3–7)	6 (4–8)	**0.013**
Conjugated bilirubin (µmol/L)	2 (1–2)	2 (1–2)	0.266	2 (1–2)	2 (2–3)	**0.024**
Iron (µmol/L)	20 (15–23)	19 (15–24)	0.698	19 (15–23)	20 (15–25)	0.278
UIBC (µmol/L)	39 (30–51)	40 (32–49)	0.998	41 (33–50)	38 (29–49)	0.355
Sodium (Na) (mmol/L)	136 (134–136)	135 (134–137)	0.575	135 (134–137)	135 (134–137)	0.868
Potassium (K) (mmol/L)	4.2 (3.9–4.3)	4.2 (4.0–4.3)	0.422	4.2 (4.0–4.3)	4.2 (4.0–4.3)	0.565
Chloride (Cl) (mmol/L)	101 (99–102)	100 (99–102)	0.896	100 (99–102)	101 (99–102)	0.370
Calcium (Ca) (mmol/L)	2.31 ± 0.08	2.31 ± 0.07	0.907	2.31 ± 0.07	2.30 ± 0.07	0.127
Magnesium (Mg) (mmol/L)	0.75 (0.72–0.78)	0.76 (0.74–0.79)	0.159	0.76 (0.73–0.79)	0.76 (0.72–0.79)	0.623
Inorganic phosphate (P) (mmol/L)	1.15 ± 0.15	1.16 ± 0.15	0.529	1.16 (1.07–1.26)	1.13 (1.05–1.26)	0.658

* The full test names of the abbreviations are available in Table 1.

**Table 3:** The reference intervals for biochemistry and hematology parameters between 11 and 14 weeks of gestation, estimated according to the CLSI C28-A3 recommendation [5]. The lower and upper reference limits are given as the 2.5th and 97.5th percentiles of the data with their respective 90% confidence intervals (CIs). When there is only one reference limit relevant, it is given as the 2.5th or 97.5th percentile of the data with the corresponding 90% CI. The reference range for non-pregnant women is recommended by the Croatian Chamber of Medical Biochemists and is harmonized for all laboratories in Croatia [12]. The values in bold represent the parameters that are clinically relevant changed according to the calculated reference change value (RCV) [6].

Analyte (Unit) *	Lower Limit for Pregnancy (90% CI)	Upper Limit for Pregnancy (90% CI)	Lower Limit Non-Pregnant	Upper Limit Non-Pregnant	RCV	%Difference Lower Reference Limit	%Difference Upper Reference Limit
RBC (×10^12^/L)	3.69 (3.64–3.80)	4.89 (4.82–5.07)	3.86	5.08	8.34	4.46	3.68
Hemoglobin (Hb) (g/L)	109 (107–112)	141 (138–147)	119	157	7.73	**8.40**	**10.51**
Hematocrit (Hct) (L/L)	0.339 (0.332–0.341)	0.429 (0.423–0.437)	0.356	0.470	8.68	4.78	**8.72**
MCV (fL)	79.0 (73.7–80.7)	96.6 (95.4–98.0)	83.0	97.2	4.23	**4.88**	0.67
MCH (pg)	24.9 (22.5–25.7)	31.8 (31.5–32.7)	27.4	33.9	3.16	**9.31**	**6.19**
MCHC (g/L)	315 (310–317)	339 (338–343)	320	345	4.55	1.72	1.74
RDW (%)	12.2 (11.7–12.4)	17.1 (16.6–18.5)	9.0	15.0	5.33	**35.56**	**14.00**
MPV (fL)	6.8 (6.6–6.9)	10.5 (10.2–11.0)	6.8	10.4	6.75	0.00	0.96
PLT (×10^9^/L)	156 (146–163)	364 (345–397)	158	424	21.22	1.58	14.15
WBC (×10^9^/L)	5.4 (5.1–5.7)	13.4 (12.8–15.1)	3.4	9.7	31.01	**58.82**	**38.14**
Neutrophile granulocytes (×10^9^/L)	3.47 (3.06–3.81)	10.23 (9.94–10.97)	2.06	6.49	39.33	**68.45**	**57.55**
Lymphocytes (×10^9^/L)	0.98 (0.88–1.07)	2.74 (2.56–2.83)	1.19	3.35	31.22	17.65	18.36
Monocytes (×10^9^/L)	0.29 (0.27–0.31)	0.99 (0.88–1.19)	0.12	0.84	40.22	**141.67**	17.26
Eosinophile granulocytes (×10^9^/L)		0.39 (0.33–0.45)		0.43	40.12		10.47
Basophile granulocytes (×10^9^/L)		0.09 (0.09–0.11)		0.06	77.26		41.67
ESR	5 (4–5)	40 (39–50)	4	24	95.49	25.00	66.67
Urea (mmol/L)	2.0 (1.7–2.0)	5.2 (4.8–5.5)	2.8	8.3	37.24	28.57	**37.95**
Creatinine (µmol/L)	35 (34–37)	65 (62–67)	49	90	14.46	**28.57**	**27.78**
Uric acid (µmol/L)	107 (94–115)	279 (265–297)	134	337	23.06	20.15	17.21
Albumin (g/L)	38.1 (37.6–38.6)	46.2 (45.7–47.3)	40.6	51.4	8.70	6.16	**10.21**
Total protein (g/L)	63 (62–63)	75 (74–76)	66	81	8.32	4.55	7.41
CRP (mg/L)		17.2 (14.2–22.5)		5.0	78.87		**244.00**
Ferritin (µg/L)	9 (8–12)	138 (121–245)	10	120	37.69	15.00	14.58
Total cholesterol (mmol/L)		6.4 (6.2–6.6)		5.0	13.36		**28.00**
Triglycerides (mmol/L)		2.6 (2.4–2.9)		1.7	46.34		**50.00**
HDL cholesterol (mmol/L)	1.3 (1.2–1.3)		1.2		16.38	8.33	
LDL cholesterol (mmol/L)		3.5 (3.4–3.9)		3.0	19.28		16.67
LD (U/L)		183 (178–204)		241	10.85		**24.07**
CK (U/L)		142 (120–218		153	33.09		7.09
AST (U/L)	10 (9–10)	24 (22–34)	8	30	24.30	**25.00**	20.00
ALT (U/L)	6 (4–7)	33 (26–37)	10	36	32.18	**40.00**	8.33
AP (U/L)	34 (31–35)	90 (80–98)	54	119	18.02	**37.04**	**24.37**
GGT (U/L)	6 (6–6)	28 (25–39)	9	35	23.43	**33.33**	20.00
AMY (U/L)	30 (25–33)	108 (96–117)	23	91	18.89	**30.43**	18.68
Total bilirubin (µmol/L)	2 (2–2)	16 (14–22)	3	20	56.45	33.33	20.00
Conjugated bilirubin (µmol/L)		5 (4–5)		5	33.32		0.00
Iron (µmol/L)	7 (5–9)	34 (33–44)	8	30	76.77	12.50	13.33
UIBC (µmol/L)	17 (7–18)	73 (69–79)	26	59	13.98	**34.62**	**23.73**
Sodium (Na) (mmol/L)	133 (131–133)	139 (138–139)	137	146	3.86	2.92	**4.79**
Potassium (K) (mmol/L)	3.7 (3.5–3.7)	4.8 (4.7–5.0)	3.9	5.1	11.58	5.13	5.88
Chloride (Cl) (mmol/L)	97 (96–97)	105 (104–105)	97	108	4.77	0.00	2.78
Calcium (Ca) (mmol/L)	2.18 (2.14–2.19)	2.45 (2.44–2.50)	2.14	2.53	6.32	1.87	3.16
Magnesium (Mg) (mmol/L)	0.67 (0.64–0.68)	0.85 (0.84–0.87)	0.65	1.05	8.77	3.08	**19.05**
Inorganic phosphate (P) (mmol/L)	0.83 (0.75–0.90)	1.41 (1.39–1.48)	0.79	1.42	22.20	5.06	0.70

* The full test names of the abbreviations are available in Table 1.

## Data Availability

The data that support the findings of this study are available from the corresponding author, V.Š.S., upon reasonable request.

## References

[1] Klajnbard A., Szecsi P.B., Colov N.P., Andersen M.R., Jørgensen M., Bjørngaard B., Barfoed A., Haahr K., Stender S. (2010). Laboratory reference intervals during pregnancy, delivery and the early postpartum period. Clin. Chem. Lab. Med..

[2] Larsson A., Palm M., Hansson L.O., Axelsson O. (2008). Reference values for clinical chemistry tests during normal pregnancy. BJOG.

[3] Barakauskas V.E., Bohn M.K., Branch E., Boutin A., Albert A., Luke S., Dittrick M., Higgins V., Adeli K., Vallance H. (2023). Mining the Gap: Deriving Pregnancy Reference Intervals for Hematology Parameters Using Clinical Datasets. Clin. Chem..

[4] Groenendijk W., Bogdanet D., Dervan L., Finn O., Islam M.N., Doheny H., Griffin T.P., Blake L., Lyons M., Kilcooley M. (2022). Reference intervals for clinical biochemistry and haematology tests during normal pregnancy. Ann. Clin. Biochem..

[5] CLSI (2008). Defining, Establishing, and Verifying Reference Intervals in the Clinical Laboratory: Approved Guideline.

[6] Fraser C.G. (2012). Reference change values. Clin. Chem. Lab. Med..

[7] Simundic A.M., Bölenius K., Cadamuro J., Church S., Cornes M.P., van Dongen-Lases E.C., Eker P., Erdeljanovic T., Grankvist K., Guimaraes J.T. (2018). Joint EFLM-COLABIOCLI Recommendation for venous blood sampling. Clin. Chem. Lab. Med..

[8] Sampson M., Ling C., Sun Q., Harb R., Ashmaig M., Warnick R., Sethi A., Fleming J.K., Otvos J.D., Meeusen J.W. (2020). A new equation for calculation of low-density lipoprotein cholesterol in patients with normolipidemia and/or hypertriglyceridemia. JAMA Cardiol..

[9] Aarsand A.K., Fernandez-Calle P., Webster C., Coskun A., Gonzales-Lao E., Diaz-Garzon J., Jonker N., Minchinela J., Simon M., Braga F. The EFLM Biological Variation Database. https://biologicalvariation.eu/.

[10] Penev M.N., Doukova-Peneva P., Kalinov K. (1996). Study on long-term biological variability of erythrocyte sedimentation rate. Scand. J. Clin. Lab. Investig..

[11] Reed A.H., Henry R.J., Mason W.B. (1971). Influence of statistical method used on the resulting estimate of normal range. Clin. Chem..

[12] Čvorišćec D., Flegar-Meštrić Z., Juretić D., Stavljenić Rukavina A., Čvorišćec D. (2007). Harmonizacija Općih Pretraga iz Područja opće Medicinske-Biokemije u Harmonizacija Laboratorijskih Nalaza u Području Opće, Specijalne i Visokodiferentne Medicinske Biokemije.

[13] Milman N., Bergholt T., Byg K.E., Eriksen L., Hvas A.M. (2007). Reference intervals for haematological variables during normal pregnancy and postpartum in 434 healthy Danish women. Eur. J. Haematol..

[14] Markus C., Flores C., Saxon B., Osborn K. (2021). Pregnancy-specific continuous reference intervals for haematology parameters from an Australian dataset: A step toward dynamic continuous reference intervals. Aust. N. Z. J. Obstet. Gynaecol..

[15] Beguin Y., Lipscei G., Oris R., Thoumsin H., Fillet G. (1990). Serum immunoreactive erythropoietin during pregnancy and in the early postpartum. Br. J. Haematol..

[16] Yang Y., Wang R., Jiang H., Hu M., Tang A., Xiang Z. (2019). Changes of iron metabolism during pregnancy and the establishment of reference intervals for pregnant Chinese women. Ann. Clin. Biochem..

[17] World Health Organization Prevalence of Anaemia in Women Aged 15–49, by Pregnancy Status (%). https://www.who.int/data/gho/indicator-metadata-registry/imr-details/4552.

[18] Beutler E., West C. (2005). Hematologic differences between African-Americans and whites: The roles of iron deficiency and alpha-thalassemia on hemoglobin levels and mean corpuscular volume. Blood.

[19] Bohn M.K., Adeli K. (2022). Physiological and metabolic adaptations in pregnancy: Importance of trimester-specific reference intervals to investigate maternal health and complications. Crit. Rev. Clin. Lab. Sci..

[20] Dockree S., Shine B., Pavord S., Impey L., Vatish M. (2021). White blood cells in pregnancy: Reference intervals for before and after delivery. EBioMedicine.

[21] Mor G., Aldo P., Alvero A.B. (2017). The unique immunological and microbial aspects of pregnancy. Nat. Rev. Immunol..

[22] Belo L., Santos-Silva A., Rocha S., Caslake M., Cooney J., Pereira-Leite L., Quintanilha A., Rebelo I. (2005). Fluctuations in C-reactive protein concentration and neutrophil activation during normal human pregnancy. Eur. J. Obstet. Gynecol. Reprod. Biol..

[23] Friis Petersen J., Friis-Hansen L.J., Jensen A.K., Nyboe Andersen A., Løkkegaard E.C.L. (2019). Early pregnancy reference intervals; 29 serum analytes from 4 to 12 weeks’ gestation in naturally conceived and uncomplicated pregnancies resulting in live births. Clin. Chem. Lab. Med..

[24] Dockree S., Brook J., James T., Shine B., Impey L., Vatish M. (2021). Pregnancy-specific reference intervals for C-reactive protein improve diagnostic accuracy for infection: A longitudinal study. Clin. Chim. Acta.

[25] Cheung K.L., Lafayette R.A. (2013). Renal physiology of pregnancy. Adv. Chronic Kidney Dis..

[26] Morton A., Teasdale S. (2022). Physiological changes in pregnancy and their influence on the endocrine investigation. Clin. Endocrinol..

[27] Guarino M., Cossiga V., Morisco F. (2020). The interpretation of liver function tests in pregnancy. Best. Pract. Res. Clin. Gastroenterol..

[28] Keiser S.D., Boyd K.W., Rehberg J.F., Elkins S., Owens M.Y., Sunesara I., Martin J.N. (2012). A high LDH to AST ratio helps to differentiate pregnancy-associated thrombotic thrombocytopenic purpura (TTP) from HELLP syndrome. J. Matern. Fetal Neonatal Med..

[29] Lu Y., Jia Z., Su S., Han L., Meng L., Tang G., Wang J., Zhang C., Xie X., Zhang Y. (2021). Establishment of trimester-specific reference intervals of serum lipids and the associations with pregnancy complications and adverse perinatal outcomes: A population-based prospective study. Ann. Med..

[30] Welge J.A., Warshak C.R., Woollett L.A. (2020). Maternal plasma cholesterol concentration and preterm birth: A meta-analysis and systematic review of literature. J. Matern. Fetal Neonatal Med..

[31] Harville E.W., Crook C.E., Bazzano L.A., Woo J.G., Burns T.L., Raitakari O., Urbina E.M., Venn A., Jacobs D.R., Steinberger J. (2021). i3C Consortium. Cardiovascular risk factors before and during pregnancy: Does pregnancy unmask or initiate risk?. J. Obstet. Gynaecol. Res..

